# Prevalence and outcomes of intrinsic capacity impairments assessed using the WHO Integrated Care for Older People (ICOPE) framework in The Gambia, South Africa, and Zimbabwe: a cross-sectional study

**DOI:** 10.1016/S2214-109X(25)00490-5

**Published:** 2026-03-01

**Authors:** Anthony Muchai Manyara, Tadios Manyanga, Momodou Jallow, Etheldreda I Yoliswa Madela, Hannah Wilson, Anya Burton, Farhanah Paruk, Chris Grundy, Lucy Gates, Isatou Drammeh, Bilkish Cassim, Rashida A Ferrand, Kate A Ward, Celia L Gregson

**Affiliations:** Global Health and Ageing Research Unit, Bristol Medical School, University of Bristol, Bristol, UK; The Health Research Unit Zimbabwe, Biomedical Research and Training Institute, Harare, Zimbabwe; Medical Research Council Unit The Gambia, London School of Hygiene & Tropical Medicine, Banjul, The Gambia; Department of Geriatrics, University of KwaZulu-Natal, Durban, KwaZulu-Natal, South Africa; Global Health and Ageing Research Unit, Bristol Medical School, University of Bristol, Bristol, UK; Global Health and Ageing Research Unit, Bristol Medical School, University of Bristol, Bristol, UK; Department of Geriatrics, University of KwaZulu-Natal, Durban, KwaZulu-Natal, South Africa; Department of Infectious Disease Epidemiology and International Health, London School of Hygiene & Tropical Medicine, London, UK; Medical Research Council Lifecourse Epidemiology Centre, University of Southampton, Southampton, UK; Medical Research Council Unit The Gambia, London School of Hygiene & Tropical Medicine, Banjul, The Gambia; Department of Geriatrics, University of KwaZulu-Natal, Durban, KwaZulu-Natal, South Africa; The Health Research Unit Zimbabwe, Biomedical Research and Training Institute, Harare, Zimbabwe; Clinical Research Department, London School of Hygiene & Tropical Medicine, London, UK; Medical Research Council Unit The Gambia, London School of Hygiene & Tropical Medicine, Banjul, The Gambia; Medical Research Council Lifecourse Epidemiology Centre, University of Southampton, Southampton, UK; Global Health and Ageing Research Unit, Bristol Medical School, University of Bristol, Bristol, UK; The Health Research Unit Zimbabwe, Biomedical Research and Training Institute, Harare, Zimbabwe

## Abstract

**Background:**

Despite rising longevity across Africa, the epidemiology of intrinsic capacity (combination of mental and physical capacities) impairments (ICIs) is understudied. We aimed to determine the prevalence of ICIs and associated sociodemographic and lifestyle factors, pain, functional limitations, and health-related quality of life (HRQoL) across three African countries.

**Methods:**

This population-based cross-sectional study recruited adults aged 40 years and older in five settings: rural (n=1052) and urban (n=1218) Gambia, rural (n=948) and urban (n=968) South Africa, and urban Zimbabwe (n=1110). Researcher-administered questionnaires and physical assessments quantified ICIs. Pain was assessed using the Brief Pain Inventory, functional limitations using Western Ontario and McMaster Universities Osteoarthritis Index (WOMAC), and HRQoL using the 5-level EQ-5D. Setting-specific associations between sociodemographic and lifestyle factors and ICIs were meta-analysed. Differences in pain, function, and HRQoL scores were computed according to ICI number.

**Findings:**

The 5296 adults had a mean age of 61·0 (SD 12·9) years; 2823 (53·3%) were female and 2473 (46·7%) were male. Prevalence of two or more ICIs was 62·4% (59·4–65·3) in rural Gambia, 57·6% (54·8–60·4) in urban Gambia, 67·6% (64·5–70·6) in rural South Africa, 72·1% (69·2–74·9) in urban South Africa, and 64·8% (61·9–67·6) in urban Zimbabwe. Having two or more ICIs was more common in those aged 55–69 years (pooled sex-adjusted odds ratio [OR] 1·79 [95% CI 1·51–2·11]) and 70 years and older (7·21 [5·01–10·37]), female individuals (pooled age-adjusted OR 1·73 [95% CI 1·35–2·22]), those who were underweight (pooled age-adjusted and sex-adjusted OR 4·72 [3·41–6·54]), those with lower wealth index (1·38 [1·20–1·58]), those with food insecurity (1·92 [1·17–3·17]), and those who reported current or former tobacco use (1·47 [1·18–1·83]). Overall mean values were 3·95 (SD 10·64) for pain score, 14·17 (18·36) for WOMAC score, and 0·83 (0·11) for HRQoL. Compared with no impairment (n=554 [10·5%]), having two or more ICIs was associated with 2·34-fold (95% CI 1·92–2·84) greater functional limitation, 2·44-fold more pain (1·86–3·20), and lower HRQoL (mean difference –0·06 [–0·07 to –0·05]).

**Interpretation:**

In both rural and urban African settings, common ICIs necessitate urgent interventions to maximise functional ability and reduce the effect on quality of life. Individuals most at risk include women, those aged ≥55 years, and people with low socioeconomic status.

**Funding:**

National Institute for Health Research (NIHR), NIHR–Wellcome Partnership for Global Health Research, and Medical Research Council Musculoskeletal Functional Ability in sub-Saharan Africa.

## Introduction

Globally, increasing longevity and falling fertility rates are creating an older population.^1^ However, inequalities in life expectancy gains exist across countries, and these added years might not equate to healthy years.^2,3^ The UN Decade of Healthy Ageing (2021–30) aims to increase life expectancy and healthy years.^4^ Healthy ageing is defined as developing and sustaining functional ability across the life course to enable wellbeing in older age.^3^ Sustaining functional ability involves maintaining intrinsic capacity (the combination of mental and physical capacities) and creating an enabling environment so people can do what matters most to them.^2,4^ To achieve this goal, WHO has proposed the Integrated Care for Older People (ICOPE) framework: an evidence-based, community-level implementation plan to screen and assess for intrinsic capacity impairments (ICIs), which then informs a personalised care plan to reverse, slow, or prevent further declines.^4,5^ A recent meta-analysis of nine studies from areas of Asia and Europe reported a 55% pooled prevalence of ICIs in participants with a mean age approaching 70 years.^6^ The ICOPE intervention improved intrinsic capacity in older adults, particularly in cognition and mood, in a meta-analysis of 32 randomised trials from different global settings.^7^ However, neither of these meta-analyses included any data from African countries.

The proportion of Africans aged 60 years and older is expected to triple by 2050,^7^ yet life expectancy will be lower compared with other global regions.^8^ Notably, Africa’s older population bears a greater prevalence of morbidity and disability compared with older populations in other regions.^9^ Despite high needs, older Africans have limited access to health care,^9^ which calls for urgent health system realignment towards older people’s needs, and healthy ageing interventions such as the ICOPE approach. However, ICOPE implementation is yet to be rolled out in Africa (although health-care worker training has begun in Botswana).^4^ Importantly, there is scant evidence on the prevalence of ICIs in African populations (based on ICOPE’s intrinsic capacity screening and assessment) to describe the magnitude of health-care needs and inform ICOPE implementation. Secondary analyses of the WHO Study on Global Ageing and Adult Health (SAGE 2007–10) survey, including Ghana and South Africa (middle-income countries), found an ICI prevalence ranging from 29% to 43% for visual impairment and 5% to 11% for depression;^10^ however, these analyses did not assess other domains such as locomotion, cognition, and vitality and did not use the ICOPE screening approaches.

This study aims to generate evidence that informs the planning of ICOPE implementation in three diverse countries: The Gambia, Zimbabwe, and South Africa. The countries represent western (The Gambia) and southern Africa regions (Zimbabwe and South Africa), and different World Bank income groups (low income [The Gambia], lower-middle income [Zimbabwe], and upper-middle income [South Africa]).^11^ Specifically, the study aims to determine the overall and domain-specific prevalence of ICIs in rural and urban settings among adults aged 40 years and older, including disaggregation by age group and sex; sociodemographic, household, and lifestyle factors associated with having more ICIs; and the association between ICIs and pain, functional limitations, and health-related quality of life (HRQoL).

## Methods

### Study design and population

The STROBE checklist for cross-sectional studies has been used to report this study (appendix 3 pp 3–4).^12^

This population-based cross-sectional study formed part of the Fractures-E3 study conducted in five sites: rural (West Kiang Demographic Surveillance Site) and urban (Sukuta and Brufut) Gambia, urban Zimbabwe (Dzivarasekwa, Mufakose, and Highfields), and rural (Mafakathini) and urban (KwaMashu) South Africa between February, 2022, and May, 2024 (data were collected between Feb 14, 2022, and Nov 28, 2023 in urban Gambia; Feb 13, 2023, and Sept 2, 2024 in rural Gambia; March 14, 2022, and March 28, 2023 in urban South Africa; June 7, 2023, and May 28, 2024 in rural South Africa; and Feb 10, 2022, and Nov 24, 2022 in urban Zimbabwe). These sites were chosen to represent typical urban and rural sites and thus be broadly representative of urban and rural sites in each country. Data were collected primarily to determine the population prevalence of vertebral fractures in older adults, although many other morbidities were studied. The study aimed to recruit 504 female and 504 male individuals in each site equally distributed (n=168) across three age strata (40–54 years, 55–69 years, and ≥70 years), generating six age–sex strata. This sample size provided at least 90% power (α=0·05) to detect with 2·5% precision an outcome with 9% prevalence (vertebral fracture), an odds ratio (OR) of 2·0 for an associated exposure with 13% prevalence (eg, HIV),^13^ ≥90% power (α=0·05) to detect with 3·6% precision a vitality ICI prevalence of 21%, and with 4·3% precision a visual ICI prevalence of 45% (as have been reported in adults aged ≥50 years in Africa).^14,15^ More details on the study are described in the published protocol.^13^

Enumeration was used to identify all residents who were eligible (lived in the study site for at least 4 weeks and aged ≥40 years) for the study.^13^ Each of the five sites was split into a set of smaller blocks, using a combination of satellite imagery and OpenStreetMap within the geographical information systems QGIS. The blocks were designed to be roughly equal in size and large enough to recruit 18–30 eligible individuals (three to five per age–sex strata). Blocks were assigned a random number, allowing a subset to be randomly selected. Within each selected block, each household was approached, enumerated, and eligible individuals were invited to participate until each age–sex stratum in the block was complete.

Ethical approval was obtained in all the study countries. In The Gambia, ethical approval was obtained from The Gambia Government–Medical Research Council Unit The Gambia, London School of Hygiene & Tropical Medicine Scientific Coordinating Committee and Ethics committee (22/04/2021 reference 22975); and the Ministry of Health (20/08/2021 reference DDHS/ AD/2021/08[MTN27]). In Zimbabwe, ethical approval was obtained from the Medical Research Council of Zimbabwe (14/07/2021 reference MRCZ/A/2706); the Biomedical Research and Training Institute (19/02/2021 reference AP161/2021); the Sally Mugabe Central Hospital (29/01/2021 reference HCHEC/ 250121/06); the University of Zimbabwe College of Health Sciences and the Parirenyatwa group of hospitals (25/02/2021); Harare City Health (27/01/2021); and the Research Council of Zimbabwe (14/07/2021 references 04246 and 04248). In South Africa, ethical approval was obtained from the University of KwaZulu-Natal’s Biomedical Research Ethics Committee (WP1 21/08/2021 BREC/00002513/2021). All participants provided written informed consent or assent before data collection.

### Procedures and definitions

Validated and standardised procedures were used to collect data and involved: researcher-administered questionnaires (sociodemographic and lifestyle [tobacco and alcohol use] characteristics), and physical assessments (eg, vision, hearing, and anthropometry).^13^ Retention and impairment of intrinsic capacities were defined as closely as possible to the WHO ICOPE assessment framework (table 1). Locomotion was assessed using the Short Physical Performance Battery (SPPB), measuring gait speed, sit-to-stand time, and tandem balance, with a score less than 10 defining impairment.^5^ Vitality was assessed using the Mini Nutritional Assessment–Short Form (MNA-SF) based on questions addressing food insecurity, weight loss, immobility, psychological stress or acute disease, neuropsychological problems, and BMI (appendix 3 p 5). Vitality was considered impaired when either the MNA-SF score was 0–7 (malnourished) or the MNA-SF score was 8–11 (risk of malnutrition) and either weight loss was self-reported in the past year or BMI was less than 19 kg/m^2^. An MNA-SF score of 12–14 (normal), or of 8–11 with no self-reported weight loss in the past year and a BMI of 19 kg/m^2^ or greater indicated retained capacity. Vision was considered impaired if the participant did not pass near or distance assessments (using tumbling Es on a tablet-installed application)^16^ or, if this was not measured, the participant self-reported difficulty seeing. Hearing was defined as impaired if the hearWHO score^17^ was equal to or below the lowest site-specific 5th percentile score of the youngest age group (aged 40–45 years; appendix 3 p 6) or if hearing loss or difficulty was self-reported. Cognition was considered impaired based on self-reported forgetfulness or inability to concentrate, a self-reported dementia diagnosis, or if the participant was taking medication for dementia. Psychological capacity was defined as impaired if someone had a Shona Symptom Questionnaire^18^ score of 8 or higher or if they self-reported auditory hallucinations or suicide ideation.

### Sociodemographic and household factors

Sociodemographic characteristics were self-reported. Marital status was defined as single (including never married, divorced, or separated), widowed, or married (including cohabiting); this was further categorised for the analysis into married or cohabiting and single or widowed. Being single or widowed was considered a risk factor category. Educational attainment was categorised as low (no formal education or just to primary level) and high (secondary or tertiary) education, with low education hypothesised as a risk factor. Employment status was categorised as employed (formal employment, informal employment, or self-employed) and unemployed. A wealth index was computed from household assets using a principal component analysis,^19,20^ with a pooled analysis across the five sites based on assets that were good measures of wealth in all three countries (more details are described elsewhere^21^). The household wealth index was categorised into low, middle, and high tertiles. Food insecurity was assessed using five yes or no questions on worrying about having enough food, variety of food, and amount of food eaten by household members in the past 4 weeks.^22^ Being food secure was defined as answering no to all questions and being food insecure as answering yes to any of the five questions.

### Lifestyle factors

Alcohol intake and tobacco use were self-reported and classified as current or former use and never used. Physical activity was assessed using the short form of the International Physical Activity Questionnaire^23^ and metabolic equivalent (MET) min per week calculated.^24^ Low physical activity was defined as less than 3000 MET min per week, with moderate or high physical activity as 3000 or more MET min per week.^25^ Weight was measured using Seca 803 scales, and height was measured using Seca 213 stadiometers. Height and weight were used to compute BMI, which was categorised as underweight (<18·5 kg/m^2^), normal weight (18·5–24·9 kg/m^2^), and overweight and obese (>25 kg/m^2^).^26^

### Function, pain, and quality of life

Functional limitations were assessed using the short form Western Ontario and McMaster Universities Osteoarthritis Index (WOMAC) Likert scale questionnaire^27^ spanning three areas: pain (ten questions), stiffness (four questions), and function (14 questions; appendix 3 pp 7–8), with area-specific and overall scores computed. A further weighted pain score based on pain reported in 45 anatomical areas was determined using the Margolis pain rating system.^28^ HRQoL was assessed using the visual analogue scale and the 5-level EQ-5D^29^ converted to a utility score based on the Zimbabwe value set.^30^

### Statistical analysis

Analyses were performed using R statistical software (version 4.3.1). Normality in the distribution of continuous variables was checked using histograms (appendix 3 pp 9–12). Normally distributed data are presented as means; skewed data are presented as medians. Categorical variables are presented as counts. Prevalence of ICIs was determined using generalised additive models (GAMs) with an estimated marginal means function. An interaction term for site was used to determine prevalence at the five sites and by age group (40–54 years, 55–69 years, ≥70 years). Associations between sociodemographic and household characteristics (age, sex, marital status, wealth index, education level, employment, and food insecurity) and lifestyle factors (BMI, tobacco use, alcohol intake, and physical activity) and having two or more ICIs were assessed for each site and then meta-analysed. The random-effects ORs and the Higgins *I*^2^ show the heterogeneity across the study sites in effect sizes.^31^ Up to 40% heterogeneity is considered negligible while up to 60% is considered moderate heterogeneity. Associations with age were adjusted for sex, whereas associations with sex were adjusted for age (continuous). All other associations were adjusted for both sex and age. Site-specific adjusted ORs and fixed-effects and random-effects pooled (across five sites) adjusted ORs are presented in forest plots. Associations between the number of ICIs (exposure) and pain and functional ability scores (outcomes) were assessed using negative binomial regression (given skewing of scores towards zero and wide variance) in the entire study population with a site interaction term. Associations between the number of ICIs and HRQoL utility score (outcome) were determined using GAMs and estimated marginal means in the entire study population, again with a site interaction term. Confidence intervals were used to infer evidence of between-group differences.

### Role of the funding source

The funders of the study had no role in study design, data collection, data analysis, data interpretation, or writing of the report.

## Results

In total, 12 185 households were visited, identifying 8840 eligible individuals, of whom 6481 were invited to participate. Of those invited, 5296 (81·7%) agreed, attended the local research clinic, and provided informed consent or assent (either directly or via proxy) to participate (appendix 3 p 13). Those who did not participate (n=1229) were most often younger (42·6% were aged 40–54 years) and comprised similar proportions of male and female individuals.

The proportion of female individuals recruited was between 51·5% and 55·1% across the five sites (table 2). The overall mean age of participants was 61·0 (SD 12·9) years (range: 40–106 years). Low educational attainment was most common in The Gambia (urban: 76·8%; rural: 89·1%). Food insecurity ranged from 31·1% in urban Zimbabwe to 68·1% in urban South Africa. Being underweight was most common in rural Gambia (15·3%), whereas 50% or more of the study population in the urban sites and rural South Africa were overweight or obese (table 2).

**Figure 1:**
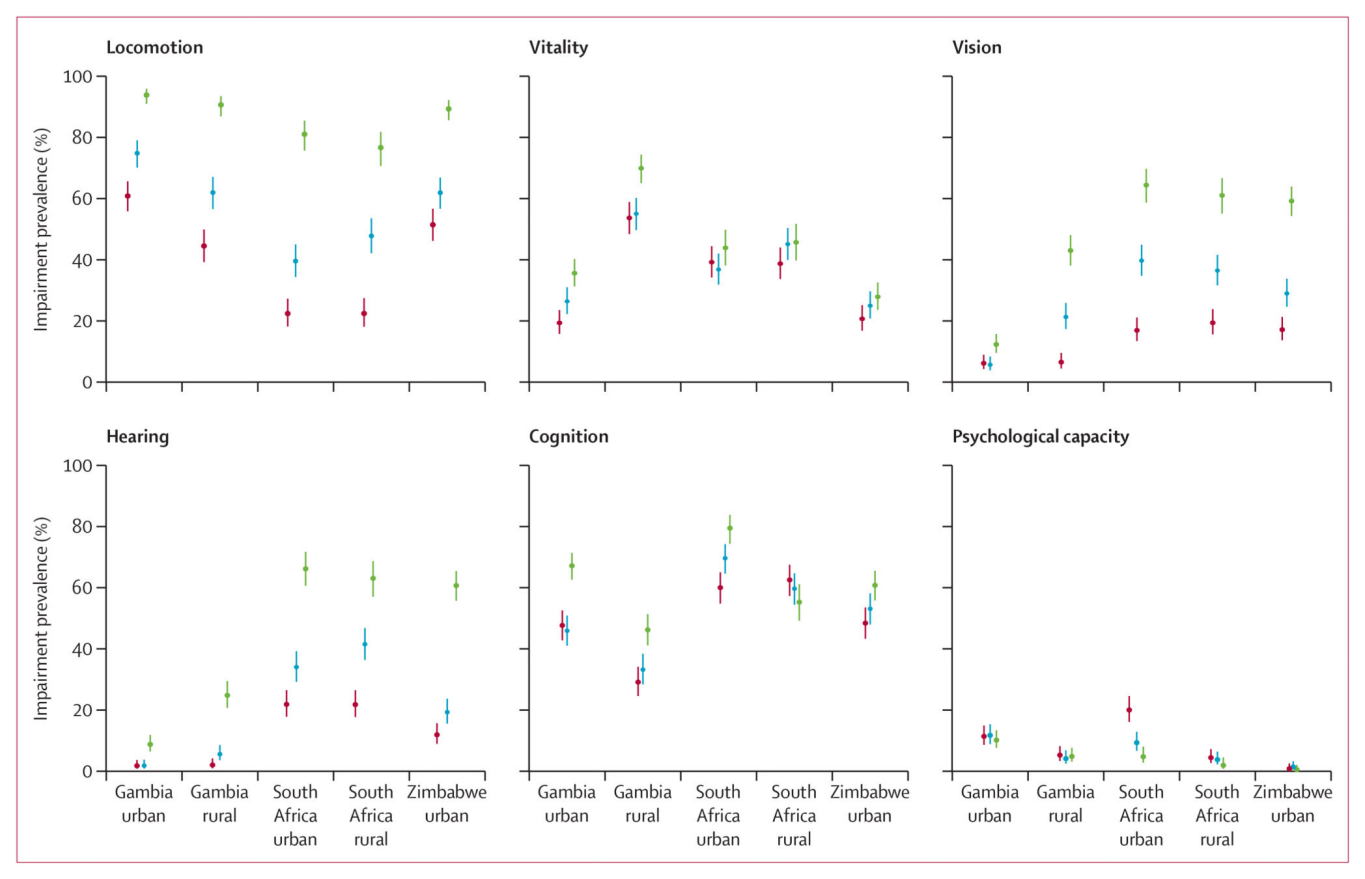
Prevalence (95% CIs) of impairments in the six intrinsic capacity domains across age groups Age groups are 40–54 years (red), 55–69 years (blue), and 70 years and older (green).

The locomotion impairment prevalence was 65·2% (95% CI 62·2–68·0) in rural Gambia, 76·5% (74·0–78·8) in urban Gambia, 46·1% (42·7–49·4) in rural South Africa, 44·9% (41·7–48·1) in urban South Africa, and 67·5% (64·7–70·2) in urban Zimbabwe. Impairment was most common in those aged 70 years and older (86·9% [85·2–88·5]) compared with those aged 55–69 years (57·7% [55·3–60·0]) and 40–54 years (41·3% [39·0–43·6]; appendix 3 pp 14–15). This pattern was seen across all sites, with urban Gambia having the highest prevalence in the three age groups (figure 1; appendix 3 p 14).

Vitality impairment prevalence was 59·9% (95% CI 56·9–62·8) in rural Gambia, 27·5% (25·1–30·1) in urban Gambia, 43·1% (40·0–46·3) in rural South Africa, 39·8% (36·7–42·9) in urban South Africa, and 24·7% (22·2–27·3) in urban Zimbabwe. Impairment was most common in the 70 years and older age group (44·1% [41·8–46·5]), with rural Gambia having the highest vitality impairment prevalence in all age groups (figure 1; appendix 3 pp 14–15).

Vision impairment prevalence was 23·9% (95% CI 21·4–26·6) in rural Gambia, 7·8% (6·4–9·5) in urban Gambia, 37·2% (34·2–40·3) in rural South Africa, 38·6% (35·6–41·7) in urban South Africa, and 35·7% (32·9–38·5) in urban Zimbabwe. Hearing impairment prevalence was 11·1% (9·4–13·2) in rural Gambia, 4·3% (3·5–5·6) in urban Gambia, 40·3% (37·3–43·5) in rural South Africa, 35·8% (32·9–38·9) in urban South Africa, and 31·3% (28·6–34·1) in urban Zimbabwe. Visual impairment increased with age and was most common in those aged 70 years and older (45·6% [95% CI 43·2–47·9]), with the highest prevalence across all age groups seen in South Africa (figure 1; appendix 3 p 14). Similarly, the 70 years and older age group had the highest prevalence of hearing impairment (41·2% [38·9–43·6]). In urban Zimbabwe and rural and urban South Africa, hearing impairment prevalence was greater than 60% in the 70 years and older age group (figure 1, appendix 3 p 14).

Cognitive impairment prevalence was 36·4% (95% CI 33·5–39·4) in rural Gambia, 54·2% (51·3–56·9) in urban Gambia, 59·6% (56·4–62·7) in rural South Africa, 69·1% (66·1–72·0) in urban South Africa, and 54·3% (51·4–57·2) in urban Zimbabwe. Overall, impairment in cognitive capacity affected 61·6% (59·3–63·9) of adults aged 70 years and older (appendix 3 p 14). Prevalence of cognitive impairment was higher than 50% across all age groups in urban and rural South Africa. Urban-dwelling South African participants aged 70 years and older had the highest prevalence of cognitive impairment (79·6% [74·5–84·0]; figure 1; appendix 3 pp 14–15).

Overall, impairments in psychological capacity were comparatively few: 4·8% (95% CI 3·6–6·2) in rural Gambia, 11·0% (9·4–12·9) in urban Gambia, 3·5% (2·5–4·9) in rural South Africa, 11·8% (9·9–14·0) in urban South Africa, and 0·9% (0·5–1·7) in urban Zimbabwe. Prevalence ranged from 4·8% (3·8–5·9) in the 70 years and older age group to 8·4% (7·2–9·8) in the 40–54 years age group (appendix 3 pp 14–15). The 40–54 years age group in urban South Africa had the highest prevalence (19·9% [16·1–24·5]; figure 1; appendix 3 p 14).

**Figure 2:**
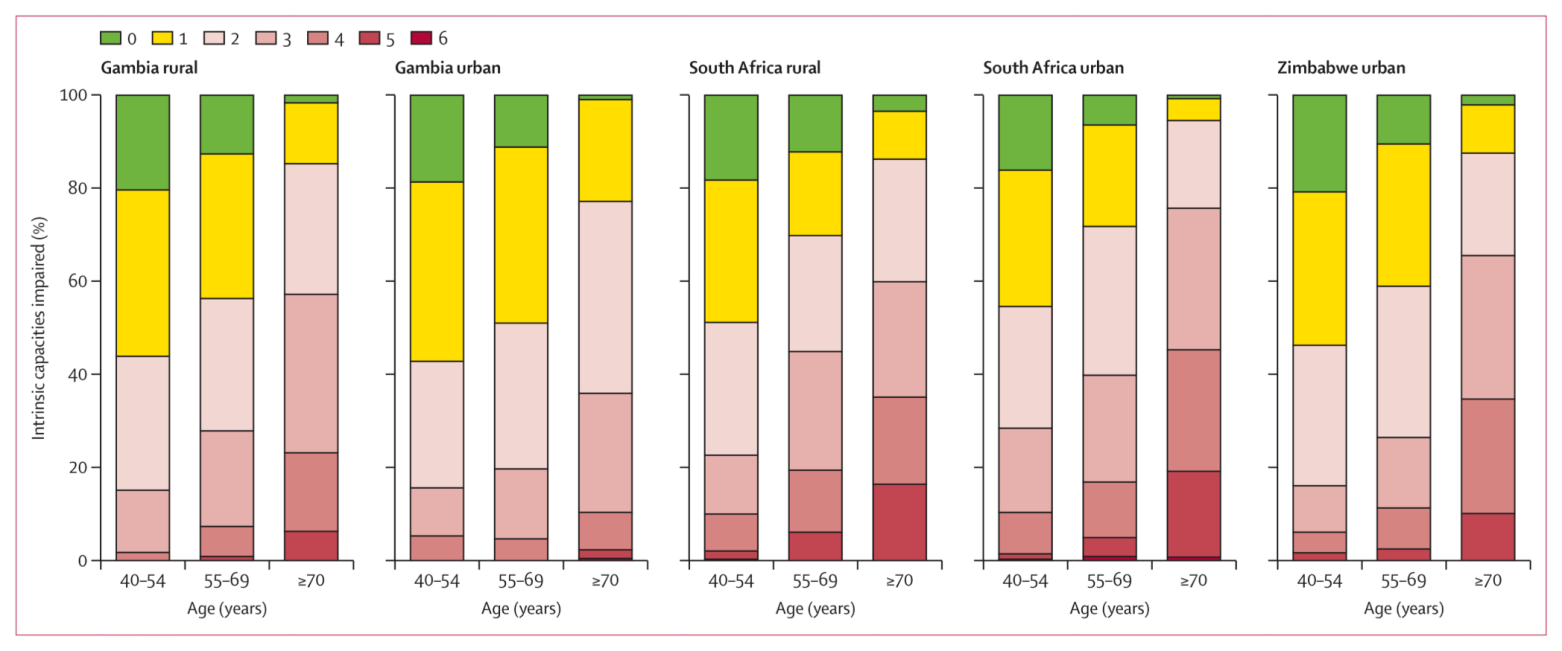
Proportion of intrinsic capacity impairments across the five study sites by three age groups Green (no impairment), yellow (one impairment), red shade (two or more impairments).

Overall, 64·5% (3416/5296) had two or more ICIs, 25·0% (1324/5296) one ICI, and only 10·5% (554/5296) had no impairments. Prevalence of two or more ICIs was 47·6% (852/1790) in the 40–54 years age group, 61·3% (1091/1779) in the 55–69 years age group, and 85·4% (1473/1725) in the 70 years and older age group (figure 2; appendix 3 p 15).

Overall, the main ICI combinations were locomotion only (632 [12·6%]), cognition and locomotion (465 [9·3%]), and cognition, locomotion, and vitality (308 [6·1%]; appendix 3 pp 16–23).

**Figure 3:**
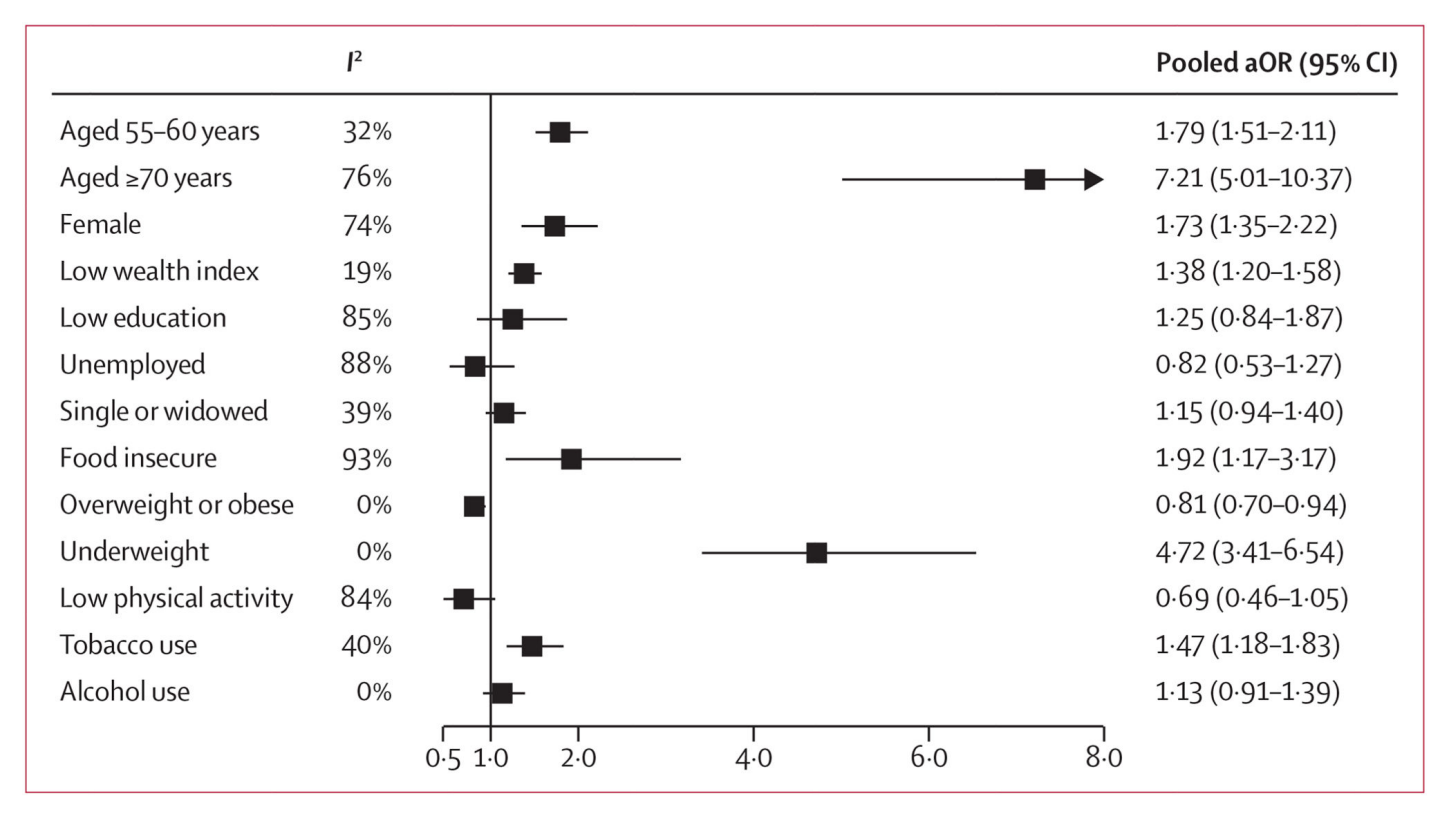
Meta-analysed associations between sociodemographic, household, and lifestyle factors and two intrinsic capacity impairments Association with age was adjusted for sex, and association with sex was adjusted for age (as a continuous variable). All other associations were adjusted for age and sex. Alcohol use models excluded The Gambia as alcohol use was very low. The reference groups are: age 40–54 years, male, middle–high wealth index, employed (formal, informal, or self-employed), married (including cohabiting), normal weight, high activity (3000 or more MET-min per week), and current or former use for tobacco and alcohol.

Having two or more ICIs was more common in those aged 55–69 years (*I*^2^ 32%; pooled OR 1·79 [95% CI 1·51–2·11) and 70 years and older (76%; 7·21 [5·01–10·37]) than in those aged 40–54 years after adjusting for sex, and it was more common in women than in men (74%; 1·73 [1·35–2·22]), after adjusting for age (figure 3; appendix 3 pp 24–25). In all study sites, locomotion capacity was more impaired in women than in men, as was cognition, albeit to a lesser extent. For other functional domains, ICIs were similar in men and women (appendix 3 p 32). After adjusting for both sex and age, low household wealth (compared with middle or high household wealth) was associated with having two or more ICIs (19%; 1·38 [1·20–1·58]), as was being food insecure (93%; 1·92 [1·17–3·17]), being underweight compared with normal weight (0%; 4·72 [3·41–6·54]), and being a current or former tobacco user compared with never using tobacco (40%; 1·47 [1·18–1·83]; figure 3; appendix 3 pp 26–29). Although unemployment versus employment was not associated with having two or more ICIs (figure 3), informal employment (5%; 1·76 [1·19–2·61]) and self-employment (0%; 1·95 [1·38–2·74]) were associated with having two or more ICIs compared with formal employment (appendix 3 pp 30, 31, 34). Being overweight or obese was associated with lower odds of having two or more ICIs compared with having a normal BMI (0·81 [0·70–0·94]; figure 3; appendix 3 p 28).

**Figure 4:**
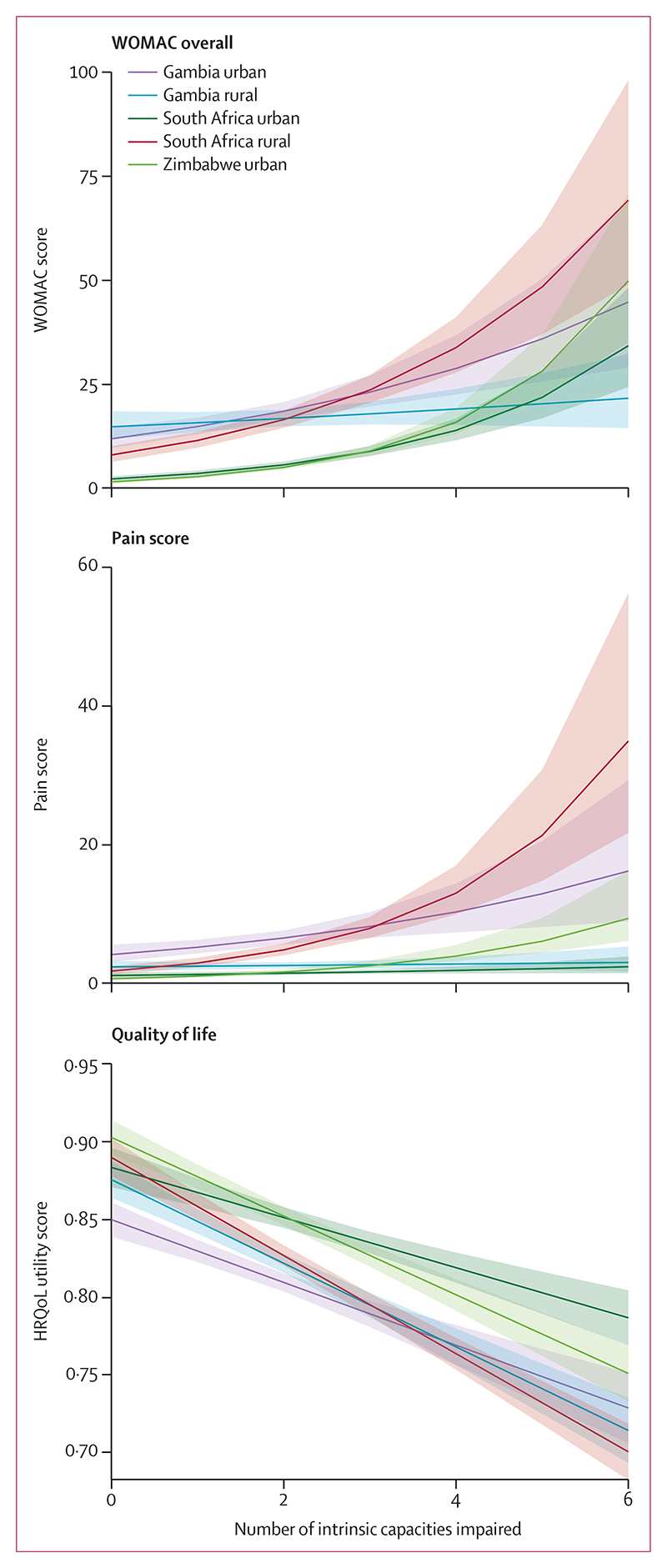
WOMAC score (A), Margolis pain score (B), and HRQoL utility score (C) Scores are according to the number of ICIs using linear modelling. Shading shows 95% CIs. HRQoL=health-related quality of life. ICI=intrinsic capacity impairment. WOMAC=Western Ontario and McMaster Universities Osteoarthritis Index.

The overall mean WOMAC score for the 554 adults with no ICIs was 7·10 (95% CI 6·18–8·15), whereas in those with two or more ICIs it was 16·6 (15·5–17·7), translating to a 2·34-fold (95% CI 1·92–2·84) higher WOMAC score, implying more knee or hip pain, stiffness, and functional limitation with more ICIs. This association was strongest in urban Zimbabwe and rural South Africa, and to a lesser extent in rural Gambia (figure 4). The overall mean pain score among people with no ICIs was 1·91 (1·58–2·32), whereas in individuals with two or more ICIs, the mean was 4·66 (4·25–5·11), equating to 2·44-fold (1·86–3·20) greater body pain. Pain increased with the number of ICIs, particularly in rural South Africa (figure 4). The overall mean HRQoL utility score was 0·88 (0·87–0·88) in those with no ICIs and 0·82 (0·81–0·82) in those with two or more ICIs, equating to a mean utility score difference of –0·06 (95% CI –0·07 to –0·05). The inverse association between cumulative ICIs and HRQoL utility scores was similar across all five study sites (figure 4). Similar patterns were seen using the visual analogue scale, in all sites except rural South Africa (appendix 3 p 34).

## Discussion

This study is the first to report on ICIs using the WHO ICOPE screening parameters across countries in Africa. ICIs were common in older adults in The Gambia, Zimbabwe, and South Africa. Among adults aged 40–54 years, almost half already had two or more impairments, increasing to more than 60% in the 55–69 years age group, and to more than 85% in the 70 years and older age group. The most common impairments were related to locomotor, cognitive, and vitality capacities. Locomotion, hearing, and vision impairments were increasingly common in adults aged 70 years and older. Psychological capacity impairment was comparatively rarely detected. In all three countries, living with more impairments was generally associated with more pain, stiffness, and functional limitation and a lower quality of life.

The high prevalence of at least one ICI in this study (89·5%; overall mean age 61·0 years) is higher than the recent meta-analyses including adults aged 60 years and older from Asia and Europe: 55% (n=52 778 adults from eight studies)^6^ and 68% (n=33 070 adults from 15 studies).^32^ The impairment prevalence in the current study is comparable to community-based surveys from rural India (999 [88%]) and the Dominican Republic (2009 [85%]),^31^ but lower than reports from China (1972 [98%]).^32^ Our study quantifies the substantial challenges to healthy ageing across three diverse countries in Africa, even at a relatively young age. Findings are consistent with an international comparison of ageing profiles (measured using T-scores), which found that African countries (only Ghana and South Africa included) had some of the poorest ageing profiles globally.^33^

The ICOPE framework presents an evidence-based approach to screen, assess, and intervene to preserve intrinsic capacity; however, implementation is only just starting in Africa.^4^ Interventions from the ICOPE framework need to be informed by context-specific evidence on health and care needs of older Africans,^9^ ideally using the framework’s screening and assessment processes. This study is the first to generate such evidence in the region. Although we studied three diverse countries, generalisability to the wider regions cannot be assumed. Results suggest that older Africans’ needs might differ by rural–urban residence even in the same country (eg, nutrition and vision needs are higher in rural Gambia than in urban Gambia). Such rural–urban differences might be due to social determinants of health (eg, educational attainment), health access (eg, to assistive devices for visual impairment), and the physical environment (eg, healthy food access).^9^ Replication of this study in other African countries and studies to understand mechanisms contributing to heterogeneity across the geographical sites beyond sociodemographic and lifestyle factors are needed. Nevertheless, interventions such as implementation of ICOPE are urgently needed to prevent, delay, and reverse ICIs. However, healthy ageing requires more than integrated care for ICIs, and should also include upstream interventions (ie, public health prevention and health promotion efforts^31^) and target the wider social determinants of health across the life course (eg, food insecurity and poverty, which were associated with impairments in the current study). Political will is needed for action.

The strengths of this study include a large sample size, rural–urban comparisons, and associations with functional outcomes and quality of life in three diverse countries. However, interpretation of findings should be made considering limitations. First, the cross-sectional nature precludes causal inference, particularly on ICIs, functional outcomes, and quality of life. Second, the study was conducted in urban settings in three countries and rural settings in two countries, and the findings may not be generalisable to other African countries. We did not have a rural Zimbabwe group, given the low population density in difficult-to-access communities, which made the rural survey impractical. Third, given that the study is a secondary analysis of data, the definition of impairment in all but the locomotion capacity did not use the exact WHO ICOPE-specific screening approach; rather, definitions were conceptualised as closely as possible to those recommended. Additionally, even for locomotor capacity, the recommended ICOPE tests and cutoffs have not been validated in African populations. Fourth, visual and hearing impairments might have been underestimated in The Gambia, given the use of only self-reported data. Fifth, the low prevalence of psychological capacity impairment observed across the sites might be underestimated, and it is possible that the assessment (Shona Symptom Questionnaire), albeit widely used, was not valid for this older population. Sixth, some associations (age 70 years and above, being female, and food insecurity) had moderate to high heterogeneity implying limited generalisability of pooled effect sizes reported to all study sites. Reassuringly, the WHO SAGE survey in Ghana and South Africa, which used the World Mental Health Survey version of the Composite International Diagnostic Interview, also reported low rates of depression.^10^ Effective identification of older people in need of ICOPE interventions will need to be based on validated screening tools; hence, there is a need for more research using locally validated approaches.

ICIs are common in Africa, particularly for locomotor and cognitive capacities, and are evident in relatively young adults (aged 40–54 years). Although there was heterogeneity in capacity impairments across countries and rural–urban settings, a common finding was the negative association between impairment burdens and functional limitations, pain, and reduced quality of life. There is an urgent need for healthy ageing interventions, which aim to preserve intrinsic capacities in African populations. Such interventions should be informed by contextual evidence and be assessed using locally validated measures.

## Figures and Tables

**Table 1:** WHO ICOPE domains and their operationalisation and definition in current study

	Operationalisation of assessments in current study	Definition of capacity
**Locomotion**		
SPPB	SPPB score (of 12) using gait speed, sit-to-stand time, and balance test	Retained capacity if SPPB score is ≥10; impaired capacity if SPPB <10
**Vitality**		
Assess nutritional status using tools such as MNA; or malnutrition universal screening tool and record BMI, arm, and calf circumferences	MNA Short-Form score using food security score, weight loss, mobility, psychological stress or acute disease, neuropsychological problems, and BMI (appendix 3 p 5)	Retained capacity if MNA score is 12–14, denoting normal nutritional status, or 8–11 (risk of malnutrition) with no weight loss in the past year and a BMI of ≥19 kg/m^2^; impaired capacity if MNA score 0–7 (malnourished), or 8–11 (risk of malnutrition) and either weight loss in the past year or a BMI of <19 kg/m^2^
**Vision**		
Use WHO simple eye chart to test near and far vision	Self-reported difficulty seeing; near and distance vision assessed using tumbling Es on tablet-installed application^16^*	Retained capacity if a pass in both near and distance vision (including with glasses or contact lenses when applicable) for both eyes or, if missing visual assessment data, self-reported no difficulty seeing; impaired capacity if a fail in either near or distance vision (including with glasses or contact lenses when applicable) or, if missing visual assessment data, a self-report of any difficulty seeing
**Hearing**		
Perform diagnostic audiometry using either of the following: pure tone diagnostic audiometry, speech audiometry, or tympanometry	Self-reported hearing loss; self-reported difficulty hearing even if using a hearing aid; quantitative assessment using hearWHO application^17^*	Retained capacity if no response to hearing loss or hearing difficulty or hearWHO score above the site-specific 5th percentile of the 40–45 years age group; impaired capacity if self-reporting hearing loss (yes or sometimes) or any difficulty hearing or hearWHO score equal to or below the site-specific 5th percentile of the 40–45 years age group (appendix 3 p 6)
**Cognition**		
MoCA or MMSE, or GPCOG	Two questions (have you been more forgetful in the past 12 months to the extent that it has significantly affected your daily life; I found myself sometimes failing to concentrate); self-reported diagnosis of dementia; or use of medication for which indication is cognitive impairment	Retained capacity if no response to both questions and not diagnosed with dementia; impaired capacity if yes response to either question or diagnosed with dementia or using medication with indication for cognitive impairment
**Psychological capacity**		
Assessment of mood using the PHQ-9 or the depression section of the MHGAP intervention guide	The Shona Symptom Questionnaire;^18^ 14 yes or no questions creates a score of 14†	Retained capacity if a score of 0–7 and no self-reported hallucinations or suicide ideation; impaired capacity if a score of 8–14 or self-reported hallucinations or suicide ideation

GPCOG=General Practitioner Assessment of Cognition. ICOPE=WHO’s Integrated Care for Older People. MHGAP=Mental Health Gap Action Programme. MMSE=Mini-Mental State Examination. MNA=Mini Nutritional Assessment. MoCA=Mini-Cog or Montreal cognitive assessment. PHQ-9=Patient Health Questionnaire. SPPB=Short Physical Performance Battery.

* In The Gambia, only self-reported data were used.

† Shona Symptom Questionnaire is a validated tool used to assess for common mental disorders in Africa.^4^

**Table 2:** Sociodemographic and lifestyle characteristics of the study population in the five sites

	Overall, n=5296	Gambia rural, n=1052	Gambia urban, n=1218	South Africa rural, n=948	South Africa urban, n=968	Zimbabwe urban, n=1110
Sex						
Female	2823 (53·3%)	547 (52·0%)	671 (55·1%)	513 (54·1%)	520 (53·7%)	572 (51·5%)
Male	2473 (46·7%)	505 (48·0%)	547 (44·9%)	435 (45·9%)	448 (46·3%)	538 (48·5%)
Age						
Age	61·0 (12·9)	61·5 (13·2)	60·6 (12·9)	60.2 (11·9)	60·1 (11·9)	62·5 (14·1)
40–54 years	1791 (33·8%)	344 (32·7%)	398 (32·7%)	340 (35·9%)	348 (36·0%)	361 (32·5%)
55–69 years	1779 (33·6%)	341 (32·4%)	386 (31·7%)	345 (36·4%)	344 (35·5%)	363 (32·7%)
≥70 years	1726 (32·6%)	367 (34·9%)	434 (35·6%)	263 (27·7%)	276 (28·5%)	386 (34·8%)
Education						
Primary or no education	2984 (56·9%)	931 (89·1%)	913 (76·8%)	504 (53·3%)	239 (24·8%)	397 (36·0%)
Secondary and above	2262 (43·1%)	114 (10·9%)	276 (23·2%)	442 (46·7%)	724 (75·2%)	706 (64·0%)
Marital status						
Married	2976 (56·3%)	868 (82·5%)	923 (76·0%)	345 (36·4%)	260 (27·0%)	580 (52·3%)
Single or widowed	2310 (43·7%)	184 (17·5%)	291 (24·0%)	602 (63·6%)	703 (73·0%)	530 (47·7%)
Employment status						
Employed*	1640 (31·0%)	656 (62·4%)	499 (41·0%)	103 (10·9%)	124 (12·8%)	258 (23·2%)
Unemployed	3647 (68·9%)	395 (37·5%)	715 (58·7%)	844 (89·0%)	841 (86·9%)	852 (76·8%)
Household wealth index						
Low	2116 (40·0%)	530 (50·4%)	420 (34·5%)	467 (49·3%)	323 (33·4%)	376 (33·9%)
Middle	2017 (38·1%)	406 (38·6%)	393 (32·3%)	314 (33·1%)	534 (55·2%)	370 (33·3%)
High	1163 (22·0%)	116 (11·0%)	405 (33·3%)	167 (17·6%)	111 (11·5%)	364 (32·8%)
Food insecurity	2350 (44·4%)	386 (36·7%)	389 (32·0%)	573 (60·5%)	657 (68·1%)	345 (31·1%)
BMI categories						
Underweight (<18·5 kg/m^2^)	369 (7·1%)	160 (15·3%)	62 (5·2%)	28 (3·1%)	44 (4·6%)	75 (6·8%)
Normal (18·5–24·9 kg/m^2^)	2163 (41·5%)	617 (59·0%)	523 (43·6%)	275 (30·0%)	271 (28·5%)	477 (43·2%)
Overweight or obese (≥25 kg/m^2^)	2683 (51·4%)	268 (25·6%)	615 (51·3%)	613 (66·9%)	636 (66·9%)	551 (50·0%)
Low physical activity	3710 (75·7%)	572 (56·0%)	1080 (92·4%)	638 (74·6%)	627 (82·0%)	793 (72·8%)
Tobacco use†	1171 (22·2%)	245 (23·3%)	200 (16·5%)	233 (24·7%)	303 (31·4%)	190 (17·1%)
Alcohol use†	839 (15·9%)	0	6 (0·5%)	249 (26·3%)	391 (40·7%)	193 (17·5%)

Data shown are n (%) or mean (SD).

* Includes formal, informal, and self-employment.

† Use refers to current or former use.

## Research in contexxt

### Evidence before this study

The number of Africans aged 60 years and older is expected to triple over the next 30 years. Sustaining intrinsic capacity (combination of mental and physical capacities) is vital for healthy ageing. WHO’s Integrated Care for Older People (ICOPE) framework, published in 2019, guides the screening and assessment of intrinsic capacity impairments (ICIs) and consequent integrated, accessible, affordable, and people-centred care for older people to improve health outcomes and health system efficiency. The framework centres on assessing and managing six domains of intrinsic capacity (locomotion, cognition, psychology, vision, hearing, and vitality) to optimise functional ability. Implementing the framework’s interventions has been reported as challenging, although implementation evidence is still limited. Implementation of interventions should be informed by evidence based on ICOPE’s screening and assessment parameters. We searched PubMed for articles that used the ICOPE framework published between Jan 1, 2019, and Jan 31, 2025, using the search term “ICOPE”. We found 184 articles; however, none were from Africa. A study using the WHO Study on Global Ageing and Adult Health (SAGE 2007–10) survey, including Ghana and South Africa, found an association between hand-grip strength and intrinsic capacity measures, although the ICOPE framework was not used.

### Added value of this study

To the best of our knowledge, the current study is the first to report on ICIs using the WHO ICOPE screening parameters across African countries. The study was done in adults aged 40 years and older in rural and urban settings in three African countries. We report the prevalence of impairments in all six ICOPE domains, identify associated factors, and quantify pain, functional limitations, and health-related quality of life. The study found a high prevalence of ICIs (≥60% of adults had two or more impairments), with women, older people, and those with a lower wealth index having higher odds of more impairments. Locomotor, cognitive, and sensory impairments were particularly common. ICIs were associated with more pain, functional limitations, and lower quality of life.

### Implications of all the available evidence

Healthy ageing interventions, focused on preserving intrinsic capacity, are urgently needed in Africa, where ICIs are common even at a relatively young age, and where they are associated with substantial pain and losses in function and quality of life. Implementing the WHO ICOPE framework for assessment and intervention offers a potential solution; however, under-resourced health-care systems, with historically limited focus on older people’s health, will need to be reconfigured. These novel data will enable informed engagement with stakeholders, including ministries of health, health workers, and older people, in identifying efficient, acceptable, and feasible strategies for implementing the ICOPE framework, particularly for vulnerable individuals.

## Data sharing

Data are available upon reasonable request. Researchers can access participant-level data via data.bris, provided ethical approvals are in place compliant with country-specific governance regulations.

## References

[1] Vollset SE, Goren E, Yuan C-W (2020). Fertility, mortality, migration, and population scenarios for 195 countries and territories from 2017 to 2100: a forecasting analysis for the Global Burden of Disease Study. Lancet.

[2] Beard JR, Officer A, de Carvalho IA (2016). The World Report on ageing and health: a policy framework for healthy ageing. Lancet.

[3] WHO (2020). Decade of Healthy Ageing: plan of action. 2021–2030.

[4] WHO (2023). Progress report on the United Nations Decade of Healthy Ageing, 2021–2023.

[5] WHO (2019). Guidance on person-centred assessment and pathways in primary care.

[6] Jayaraj V, Gnanasekaran S, Vb Y (2024). Estimating the prevalence of intrinsic capacity decline: a systematic review and meta-analysis using WHO’s integrated care of older people (ICOPE) screening tool. Arch Gerontol Geriatr Plus.

[7] He W, Aboderin I, Adjaye-Gbewonyo D (2020). Africa aging.

[8] Vollset SE, Ababneh HS, Abate YH (2024). Burden of disease scenarios for 204 countries and territories, 2022–2050: a forecasting analysis for the Global Burden of Disease Study 2021. Lancet.

[9] Aboderin IAG, Beard JR (2015). Older people’s health in sub-Saharan Africa. Lancet.

[10] Arokiasamy P, Selvamani Y, Jotheeswaran AT, Sadana R (2021). Socioeconomic differences in handgrip strength and its association with measures of intrinsic capacity among older adults in six middle-income countries. Sci Rep.

[11] World Bank (2023). World Bank income groups.

[12] von Elm E, Altman DG, Egger M, Pocock SJ, Gøtzsche PC, Vandenbroucke JP (2007). Strengthening the Reporting of Observational Studies in Epidemiology (STROBE) statement: guidelines for reporting observational studies. BMJ.

[13] Burton A, Drew S, Cassim B (2023). Fractures in sub-Saharan Africa: epidemiology, economic impact and ethnography (Fractures-E3): study protocol. Wellcome Open Res.

[14] Manyara AM, Manyanga T, Naidoo S (2025). Nutrition outcomes and interventions in older people in Africa: a systematic umbrella and scoping review. Nutr Rev.

[15] Xulu-Kasaba ZN, Kalinda C (2022). Prevalence of blindness and its major causes in sub-Saharan Africa in 2020: a systematic review and meta-analysis. Br J Vis Impair.

[16] Rono H, Bastawrous A, Macleod D (2021). Effectiveness of an mHealth system on access to eye health services in Kenya: a cluster-randomised controlled trial. Lancet Digit Health.

[17] De Sousa KC, Smits C, Moore DR, Chada S, Myburgh H, Swanepoel W (2022). Global use and outcomes of the hearWHO mHealth hearing test. Digit Health.

[18] Patel V, Simunyu E, Gwanzura F, Lewis G, Mann A (1997). The Shona Symptom Questionnaire: the development of an indigenous measure of common mental disorders in Harare. Acta Psychiatr Scand.

[19] Bikos LH (2022). ReCentering Psych Stats: psychometrics.

[20] Revelle W (2015). Package ‘psych’. The comprehensive R archive network.

[21] Manyara AM, Jallow M, Manyanga T (2025). Measuring wealth in rural and urban Africa: findings and recommendations from a multi-country study. medRxiv.

[22] Coates J, Swindale A, Bilinsky P Household Food Insecurity Access Scale (HFIAS) for measurement of food access: indicator guide.

[23] Lee PH, Macfarlane DJ, Lam TH, Stewart SM (2011). Validity of the international physical activity questionnaire short form (IPAQ-SF): a systematic review. Int J Behav Nutr Phys Act.

[24] Forde C Scoring the international physical activity questionnaire (IPAQ).

[25] Yin X, Zhang T, Zhang Y, Man J, Yang X, Lu M (2022). The global, regional, and national disease burden of breast cancer attributable to low physical activity from 1990 to 2019: an analysis of the Global Burden of Disease Study 2019. Int J Behav Nutr Phys Act.

[26] Weir CB, Jan A (2025). BMI classification percentile and cut off points.

[27] McConnell S, Kolopack P, Davis AM (2001). The Western Ontario and McMaster Universities Osteoarthritis Index (WOMAC): a review of its utility and measurement properties. Arthritis Rheum.

[28] Margolis RB, Tait RC, Krause SJ (1986). A rating system for use with patient pain drawings. Pain.

[29] Herdman M, Gudex C, Lloyd A (2011). Development and preliminary testing of the new five-level version of EQ-5D (EQ-5D-5L). Qual Life Res.

[30] Jelsma J, Hansen K, De Weerdt W, De Cock P, Kind P (2003). How do Zimbabweans value health states?. Popul Health Metr.

[31] Prince MJ, Acosta D, Guerra M (2021). Intrinsic capacity and its associations with incident dependence and mortality in 10/66 Dementia Research Group studies in Latin America, India, and China: a population-based cohort study. PLoS Med.

[32] Cao X, Yi X, Chen H, Tian Y, Li S, Zhou J (2024). Prevalence of intrinsic capacity decline among community-dwelling older adults: a systematic review and meta-analysis. Aging Clin Exp Res.

[33] Sanchez-Niubo A, Forero CG, Wu YT (2021). Development of a common scale for measuring healthy ageing across the world: results from the ATHLOS consortium. Int J Epidemiol.

